# Efficacy and Safety of Chinese Herbal Medicine on Ovarian Cancer After Reduction Surgery and Adjuvant Chemotherapy: A Systematic Review and Meta-Analysis

**DOI:** 10.3389/fonc.2019.00730

**Published:** 2019-08-16

**Authors:** Rongyun Wang, Qiuhua Sun, Fang Wang, Yuan Liu, Xiang Li, Tianhui Chen, Xiaoke Wu, Huijuan Tang, Mengyun Zhou, Shuzhi Zhang, Yun Xiao, Weijia Huang, Chi Chiu Wang, Lu Li

**Affiliations:** ^1^School of Nursing, Zhejiang Chinese Medical University, Hangzhou, China; ^2^School of Medicine, Shanghai Jiaotong University, Shanghai, China; ^3^The First School of Clinical Medicine, Lanzhou University, Lanzhou, China; ^4^Department of Obstetrics and Gynecology, First Affiliated Hospital, Heilongjiang University of Chinese Medicine, Harbin, China; ^5^Group of Molecular Epidemiology and Cancer Precision Prevention, Zhejiang Academy of Medical Sciences, Hangzhou, China; ^6^School of Medicine, Second Affiliated Hospital, Zhejiang University, Hangzhou, China; ^7^College of Basic Medical Sciences, Zhejiang Chinese Medical University, Hangzhou, China; ^8^Department of Obstetrics and Gynaecology, The Chinese University of Hong Kong, Sha Tin, Hong Kong; ^9^Institute of Chinese Medicine, The Chinese University of Hong Kong, Sha Tin, Hong Kong

**Keywords:** meta-analysis, ovarian cancer, Chinese herbal medicine, efficacy, safety

## Abstract

**Background:** Ovarian cancer (OvC) is a malignant tumor which invades ovarian epithelium and interstitium. Reduction surgery combined with adjuvant chemotherapy is standard treatment for OvC patients, but the adverse effects due to chemotherapy still remains a major problem. While Chinese herbal medicine (CHM) therapy has a unique therapeutic effect to reduce side effects of chemotherapy by boosting immune system, the evidence of CHM in the treatment of OvC patients are limited.

**Objective:** We conducted a systematic review to evaluate the efficacy and safety of CHM in the treatment of OvC after reduction surgery and adjuvant chemotherapy.

**Method:** Chinese National Knowledge Infrastructure (CNKI) and PubMed up to Dec 31st 2018 were searched to identify relevant studies. Only randomized controlled trials (RCTs) were included, and there was no limitation on language of the publication. Data were extracted from all included studies and meta-analysis was performed with Review Manager 5.3. Study quality was assessed and pooled risk ratios (RR) or mean difference (MD) with 95% CIs were used to evaluate the efficacy and safety of CHM.

**Results:** A total of 18 RCTs involving 975 participants were included. There was no placebo, no treatment and CHM alone. Compared with Western Medicine (WM) alone, Chinese herbal Medicine combined with WM (CHM-WM) significantly improved TCM syndromes and symptoms, KPS scores, CD4 counts, CA125 levels, and 3-years survival rate (*P* < 0.05). Incidences of gastrointestinal reactions, marrow depression, urinary system symptoms were significantly lower in CHM-WM group than in WM group (*P* < 0.01). There was no significant difference in CD3 counts, CD8 counts, quality of life, liver function, and peripheral neuropathy between the two groups (*P* > 0.05).

**Conclusion:** The systematic review indicated that CHM combined with WM is effective and safe as a treatment for OvC patients after reduction surgery and adjuvant chemotherapy. However, more high-quality and large-scale RCTs are needed to confirm the efficacy and safety of CHM intervention.

Ovarian Cancer (OvC) is a gynecological malignancy with high prevalence in women aged 50–70. It accounts for about 20% of all female reproductive cancers (1). Although the morbidity in OvC is lower than that in cervical and endometrial cancers, OvC has the highest mortality amongst the three, which is the leading cause of cancer-associated death in women (2). Owing to lack of typical symptoms and early detection methods, diagnosis is often belated. Fewer than one-half of patients can survive beyond 5 years after diagnosis (3). Over 60–70% of patients are diagnosed at advanced stage. In terminal stage, patients always suffer from severe abdominal pain and distension due to peritoneal metastasis.

The National Comprehensive Cancer Network (NCCN) Guidelines recommend removal of the ovary and fallopian tubes as an initial treatment for OvC to patients with FIGO stage I and/or low-grade invasive carcinoma, and debulking surgery for patients with FIGO stage II-IV (4). Adjuvant treatments are necessary to minimize recurrence of OvC, which may include radical surgery (such as hysterectomy, unilateral salpingo-oophorectomy, etc.), radiotherapy (such as high-energy x-rays, etc.), chemotherapy (such as carboplatin plus paclitaxel regimen, cisplatin plus cyclophosphamide regimen, etc.), hormone therapy (such as tamoxifen, letrozole, etc.), tumor-targeted therapy (such as monoclonal antibody therapy, bevacizumab, etc.), and/or Chinese herbal medicine (CHM) (such as Bushenxiaozheng decoction, Lichongshensui decoction, etc.). Most postoperative patients suffer from constitutional debility and other surgery-related complications. While chemotherapy kills both tumor cells and normal cells, leading to many adverse effects, such as marrow depression, gastrointestinal reactions (nausea, vomiting), neurotoxicity, so on. In recent years, a growing number of clinical studies showed CHM could alleviate chemotherapy-related side effects and improves human immunity, which can be a supporting therapy of the adjuvant treatment for OvC (5).

## Objective

The systematic review aimed to assess the efficacy and safety of CHM for ovarian cancer after reduction surgery and adjuvant chemotherapy.

## Materials and Methods

### Inclusion and Exclusion Criteria

#### Inclusion Criteria

(1) Patients were confirmed with diagnosis of OvC at FIGO stage II-IV by surgery and pathology;
(2) The tumors were primary, and the patients included should not have any other untreated malignant tumors simultaneously;
(3) OvC patients carried out reduction surgery and adjuvant platinum-based chemotherapy;
(4) No contraindications to chemotherapy, including bone marrow depression, fever, liver and renal dysfunction, blood picture, and electrocardiogram abnormalities;
(5) Study intervention started with comparable baseline;
(6) Life expectancy was longer than 6 months for observation;
(7) No serious diseases in major organs and systems;
(8) Patients participated in the trial voluntarily.

#### Exclusion Criteria

(1) Not meeting the diagnostic criteria;
(2) Allergic to drugs;
(3) Nursing women;
(4) With mental diseases not easy or refuse to cooperate;

Shedding cases, such as subjects with poor compliance, were asked to quit the study, etc.

#### Types of Research

Only randomized controlled trials (RCTs) were included.

#### Interventions and Comparison

(1) CHM vs. placebo;
(2) CHM vs. no treatment;
(3) CHM vs. WM;
(4) CHM combined with WM vs. WM alone; and
(5) CHM vs. other interventions (bed rest, nutritional support, etc.).

### Literature Search

#### Database

We performed a comprehensive search from CNKI and PubMed databases for all the potentially eligible trials of CHM for OvC. All databases were searched from 31st January, 1966 to Dec 31st, 2018.

#### Search Strategy

Keywords for the search included “Chinese Medicine,” “Chinese Herbal Medicine,” “Traditional Chinese Medicine,” and “Ovarian Cancer.” For the CNKI database, the key words were searched in Chinese characters and Pinyin. There was no limitation on the languages.

#### Data Extraction

Based on a pre-designed and standardized data collection form, two authors (WRY & LL) reviewed the titles and abstracts of all the clinical studies independently for study inclusion. Subsequently, two authors read the full texts for study inclusion. Any non-conformity would be solved by discussion with the third author (CTH) to make a consensus. The following information was extracted from the included studies: first author, year, sample size, study design, baseline information, randomization, therapeutic outcomes, and adverse effects.

#### Quality Assessment

Assessment of methodological quality was conducted in accordance with Cochrane Reviewers' Handbook 5.0, including the randomization method, allocation concealment, description of inclusion criteria, evaluation on the curative effect with blinding, description of withdrawal and loss of follow-up, baseline consistency, and whether the intention-to-treatment (ITT) analysis was performed.

#### Data Synthesis and Analysis

We processed and analyzed the data using the Review Manager software (Revman 5.3, provided by the Cochrane Collaboration). Random-effects models were used to calculate pooled effects. Fixed-effect models were used for combining data where it was reasonable to assume that studies were estimating the same underlying treatment effect: i.e., where trials were examining the same intervention, and the trials' populations and methods were judged sufficiently similar. Dichotomous data were presented as pooled Risk Ratio (RR) with 95% confidence intervals (95% CIs), while continuous data were presented as Mean Difference (MD) with 95% CIs. We performed forest plot and funnel plot analysis to test heterogeneity, and assess reporting biases. *P* < 0.05 was considered statistically significant.

Heterogeneity was assessed through the *I*^2^ statistic, which estimates the fraction of variance that is due to heterogeneity and by Q test. The level of significance for the Q test was defined as *P* < 0.10.

## Results

### Literature Search

**Figure 1:**
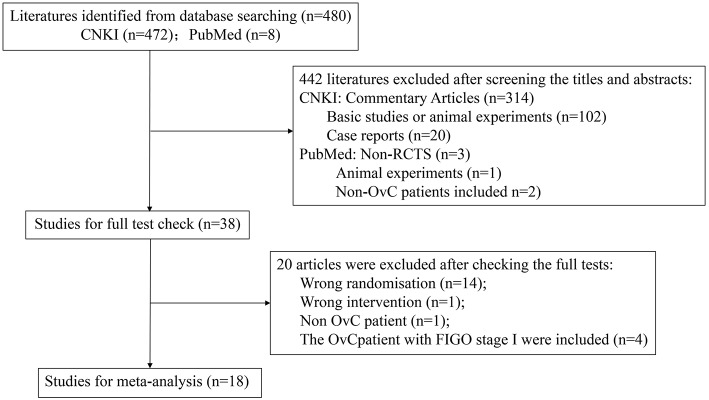
Study inclusion and exclusion.

480 clinical studies were identified in the literature search. After screening the titles and abstracts, 38 RCTs were selected initially according to the inclusion, and exclusion criteria. Subsequently, full texts of these studies were further reviewed, 20 studies were further excluded, and 18 studies were finally included for meta-analysis (6–23). Amongst these excluded studies, 14 studies applied wrong randomization (24–37), 4 trials reported only recruit FIGO stage II-IV patients but mixed with FIGO stage I patients in their outcome reports (38–41), 1 study used wrong intervention (42), and 1 study included non OvC patient (43). Besides, the subjects and study design of 2 trials (12, 18) were same, but the outcomes were different. We failed to get the responses and clarifications from the original authors. After discussion, we included all of these studies. Figure 1 summarizes the process of the study selection.

### Characteristics and Quality of the Studies

Table 1 shows a summary and quality assessment of all included studies. In total 18 studies with 975 patients were analyzed, whereas 488 patients were from study group (treated with combined CHM and WM), and the other 487 patients were from control group (treated with WM alone). There was no study group treated with CHM alone, and no placebo and no treatment in control group. CHM included Shenlingbaizhu decoction, Guizhifuling capsules, so on, where WM included Docetaxel, Cisplatin, so on. There were no significant differences in ages, BMI, clinical stages, pathological types, histological grade between the groups (*P* > 0.05). The baselines of patients' information between groups were similar, but blinding, allocation concealment and ITT were not reported in all studies.

**Table 1:** Summary of characteristics of included studies.

**Study ID**	**T/C (*n*)**	**Interventions**	**Control**	**Follow-up**	**Baseline similarity**	**Randomization**	**Blinding**	**Drop-off (%)**
Chen (6)	20/20	1. CHM fomula, 200 ml, po, BID, 8 weeks; 2. Docetaxel, 70–100 mg/m^2^, ivgtt, day 1 and day 8, 21 days^*^2 courses; 1. Cisplatin, 60 mg/m^2^, ivgtt, day 1 and day 8, 21 days^*^2 courses	1. Docetaxel, 70–100 mg/m^2^, ivgtt, day 1 and day 8, 21 days^*^2 courses; 2. Cisplatin, 60 mg/m^2^, ivgtt, day 1 and day 8, 21 days^*^2 courses	Not reported	Comparable (*P* > 0.05)	Randomized	Not reported	0
Chen (7)	30/29	1. CHM fomula, 100 ml, po, BID, 18 days^*^2 courses; 2. Taxol, 175 mg/m^2^, ivgtt, day 1, 21 days^*^2 courses; 3. Carboplatin, 300 mg/m^2^, ivgtt, Day 2, 21 days^*^2 courses	1. Taxol, 175 mg/m^2^, ivgtt, Day 1, 21 days^*^2 courses; 2. Carboplatin, 300 mg/m^2^, ivgtt, Day 2, 21 days^*^2 courses	Not reported	Comparable (*P* > 0.05)	Number randomized	Not reported	1.7
Cheng and Zhang (8)	31/31	1. CHM fomula, po, BID, 21 days; 2. Pemetrexed, 500 mg/m^2^, ivgtt, Day 1, 1 course; 3. Carboplatin, AUC = 5, ivgtt, Day 1, 1 course	1. Pemetrexed, 500 mg/m^2^, ivgtt, Day 1, 1 course; 2. Carboplatin, AUC = 5, ivgtt, Day 1, 1 course	1 month	Comparable (*P* > 0.05)	Number randomized	Not reported	0
Guo (9)	27/27	1. Puerarin injection, 400 mg, ivgtt, QD, 21 days; 2. Docetaxel, 75 mg/m^2^, ivgtt, Day 1, day 8, and day 15, 21 days^*^1–6 courses; 3. Cisplatin, 30 mg/m^2^, ivgtt, Day 1–3, 21 days^*^1–6 courses	1. Docetaxel, 75 mg/m^2^, ivgtt, Day 1, day 8, and day 15, 21 days^*^1–6 courses; 2. Cisplatin, 30 mg/m^2^, ivgtt, Day 1–3, 21 days^*^1–6 courses	Not reported	Comparable (*P* > 0.05)	Randomized	Not reported	0
Han et al. (10)	25/25	1. CHM fomula, 200 ml, po, BID, 21 days^*^3 courses; 2. Taxol, 135 mg/m^2^, ivgtt, Day 1, 21 days^*^3 courses; 3. Carboplatin, 300–500 mg/m^2^, ivgtt, Day 2, 21 days^*^3 courses	1. Taxol, 135 mg/m^2^, ivgtt, Day 1, 21 days^*^3 courses; 2. Carboplatin, 300–500 mg/m^2^, ivgtt, Day 2, 21 days^*^3 courses	Not reported	Comparable (*P* > 0.05)	Randomized	Not reported	0
Hao (11)	20/21	1. CHM fomula, 200 ml, PO, BID, 3 weeks; 2. Taxol, 135 mg/m^2^, ivgtt, 1 course; 3. Carboplatin, AUC = 5, ivgtt, 1 course;	1. Taxol, 135 mg/m^2^, ivgtt, 1 course; 2. Carboplatin, AUC = 5, ivgtt, 1 course;	Not reported	Comparable (*P* > 0.05)	Randomized	Not reported	0
Li (12)	19/20	1. CHM Capsule, 0.31 g ^*^3, po, TID, 21 days^*^2 courses; 2. Earthworm, 10 g, po, QD, 21 days^*^2 courses; 3. Taxol, 135 mg/m^2^, ivgtt, Day 1, 21 days^*^2 courses; 4. Carboplatin, AUC = 5, ivgtt, Day 1, 21 days^*^2 courses	1. Taxol, 135 mg/m^2^, ivgtt, Day 1, 21 days^*^2 courses; 2. Carboplatin, AUC = 5, ivgtt, Day 1, 21 days^*^2 courses	Not reported	Comparable (*P* > 0.05)	Randomized	Not reported	2.5
Li (13)	30/30	1. CHM fomula, 200 ml, po, BID, 6 weeks; 2. Taxol, 135 mg/m^2^, ivgtt, Day 1, 21 days^*^2 courses; 3. Carboplatin, 75 mg/m^2^, ivgtt, Day 1, 21 days^*^2 courses	1. Taxol, 135 mg/m^2^, ivgtt, Day 1, 21 days^*^2 courses; 2. Carboplatin, 75 mg/m^2^, ivgtt, Day 1, 21 days^*^2 courses	Not reported	Comparable (*P* > 0.05)	Randomized	Not reported	0
Liu et al. (14)	30/30	1. CHM fomula, 150 ml, po, BID, 4 weeks; 2. Taxol, 135 mg/m^2^, ivgtt, Day 1, 1 course; 3. Cisplatin, 75 mg/m^2^, ivgtt, Day 1, 1 course	1. Taxol, 135 mg/m^2^, ivgtt, Day 1, 1 course; 2. Cisplatin, 75 mg/m^2^, ivgtt, Day 1, 1 course	Not reported	Comparable (*P* > 0.06)	Randomized	Not reported	0
Ma (15)	15/15	1. CHM fomula, 150 mg, po, BID, 8 weeks; 2. Docetaxel, 135 mg/m^2^, ivgtt, Day 1, 21 days^*^2 courses; 3. Cisplatin, 75 mg/m^2^, ivgtt, Day 1,21 days^*^2 courses	1. Docetaxel, 135 mg/m^2^, ivgtt, Day 1, 21 days^*^2 courses; 2. Cisplatin, 75 mg/m2, ivgtt, Day 1,21 days^*^2 courses	Not reported	Comparable (*P* > 0.05)	Randomized	Not reported	0
Mei (16)	20/20	1. TCM fomula, 250 ml, po, BID, 6 weeks; 2. Taxol, 135 mg/m^2^, ivgtt, Day 1, 3 weeks^*^2 courses; 3. Cisplatin, 75 mg/m^2^, ivgtt, Day 1, 3 weeks^*^2 courses	1. Taxol, 135 mg/m^2^, ivgtt, Day 1, 3 weeks^*^2 courses; 2. Cisplatin, 75 mg/m^2^, ivgtt, Day 1, 3 weeks^*^2 courses	Not reported	Comparable (*P* > 0.05)	Randomized	Not reported	0
Qiu (17)	20/20	1. CHM fomula, 100 ml, PO, BID, 21 days; 2. Taxol, 135 mg/m^2^, ivgtt, Day 1, 1 course; 3. Carboplatin, the dose according to patient, ivgtt, Day 2, 1 course;	1. Taxol, 135 mg/m^2^, ivgtt, Day 1, 1 course; 2. Carboplatin, the dose according to patient, ivgtt, Day 2, 1 course;	Not reported	Comparable (*P* > 0.05)	Number table randomized	Not reported	0
Zhao (18)	19/20	1. CHM Capsule, 0.31 g ^*^3, po, TID, 21 days^*^2 courses; 2. Earthworm, 10 g, po, QD, 21 days^*^2 courses; 3. Taxol, 135 mg/m^2^, ivgtt, Day 1, 21 days^*^2 courses; 4. Carboplatin, AUC = 5, ivgtt, Day 1, 21 days^*^2 courses	1. Taxol, 135 mg/m^2^, ivgtt, Day 1, 21 days^*^2 courses; 2. Carboplatin, AUC = 5, ivgtt, Day 1, 21 days^*^2 courses	Not reported	Comparable (*P* > 0.05)	Number table randomized	Not reported	2.5
Jia (19)	42/42	1. CHM fomula, 200 ml, po, BID, 6 months; 2. Day 1–3, Docetaxel (60 mg/m^2^) + Cisplatin (50 mg/m^2^), ivgtt, From day 4, Docetaxel (90 mg/m^2^) + Cisplatin (60 mg/m^2^), IPT^#^, Once or twice a day, 6 months	Days 1–3, Docetaxel(60 mg/m^2^) +Cisplatin (50 mg/m^2^), ivgtt, From day 4, Docetaxel (90 mg/m^2^) + Cisplatin (60 mg/m^2^), IPT^#^, Once or twice a day, 6 months	3 years	Comparable (*P* > 0.05)	Randomized	Not reported	0
Yi et al. (20)	30/30	1. CHM fomula, 100 ml, po, BID, 4 weeks; 2. IL-2 (2 million U) + 0.9% NaCl (20 ml), Intraperitoneal perfusion, QW, 4 weeks	IL-2 (2 million U) + 0.9%NaCl(20 ml), intraperitoneal perfusion, QW, 4 weeks	Not reported	Comparable (*P* > 0.05)	Number table randomized	Not reported	0 0
Mao et al. (21)	36/35	1. CHM fomula 150 ml, po, BID, 30 days^*^6; 2. Matrine Injection (4 ml) + Shenmai injection (50 ml), ivgtt, Day 1–9, Once per months, 6 months	Matrine injection (4 ml) + Shenmai injection (50 ml), ivgtt, Day 1–9, Once per months, 6 months	6 months	Comparable (*P* > 0.05)	Randomized	Not reported	0
Xu (22)	40/40	1. TCM fomula, 100 ml, po, BID, 5 weeks; 2. Taxol, 135 mg/m^2^, ivgtt, Day 1, 21 days^*^3 courses; 3. Cisplatin, 75 mg/m^2^, ivgtt, Day 1, 21 days^*^3 courses 4. Taxol + Cisplatin, 60 mg/m^2^, IPT^#^, once per course, 21 days^*^3 courses	1. Taxol, 135 mg/m^2^, ivgtt, Day 1, 21 days^*^3 courses; 2. Cisplatin, 75 mg/m^2^, ivgtt, Day 1, 21 days^*^3 courses 3. Taxol + Cisplatin, 60 mg/m^2^, IPT^#^, once per course, 21 days^*^3 courses	3 months	Comparable (*P* > 0.05)	Randomized	Not reported	0
Zhang (23)	34/32	1. CHM fomula, Accupoint application, QD, 1 week; 2. Normal nursing	Normal nursing	Not reported	Comparable (*P* > 0.05)	Number table randomized	Not reported	0

# *IPT, intraperitoneal perfusion chemotherapy*.

### Efficacy and Safety

Outcomes of efficacy and safety were separately analyzed as below and summarized as in Supplementary Table 1. Comparisons and meta-analysis were only available and performed between combined Chinese herbal Medicine and Western Medicine (CHM-WM) group and Western Medicine alone (WM) group.

#### Efficacy

##### Syndromes and symptoms

**Figure 2:**
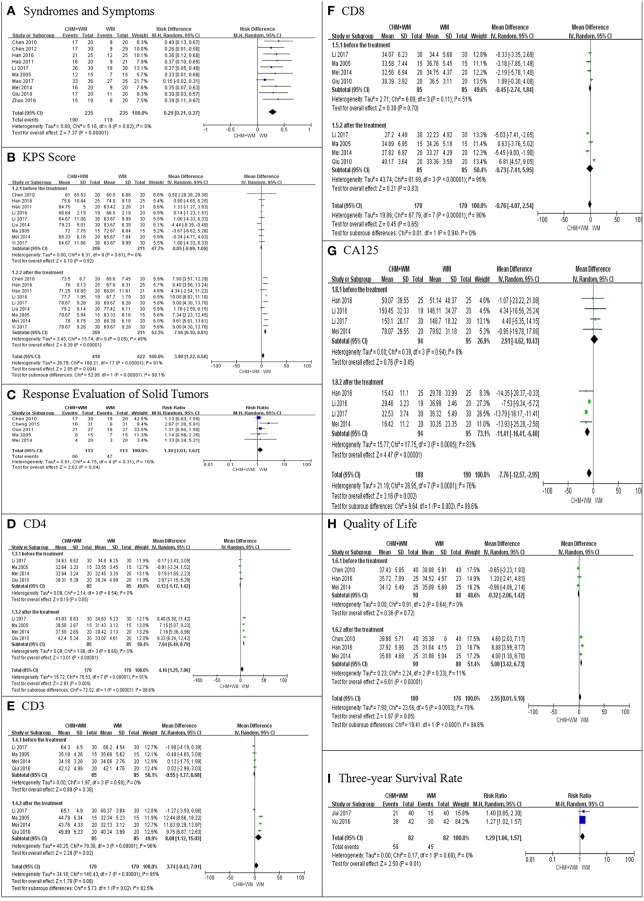
Meta-analysis on efficacy. **(A–I)** showed the comparisons and meta-analyses on efficacy between CHM-WM group and WM group. The I2 statistic described the percentage of total variation across studies that was due to heterogeneity rather than chance. CI indicated the confidence interval. Dichotomous data were presented as pooled Risk Ratio (RR) with 95% confidence intervals (95% CIs), while continuous data were presented as Mean Difference (MD) with 95% CIs.

Ten trials (6, 7, 10, 11, 13, 15–18, 21) evaluated the efficacy in the improvement of TCM syndromes and symptoms (such as poor appetite, fatigue, etc.) between the two groups. Meta-analysis showed that the symptoms were significantly improved in CHM-WM group when compared with WM group (RR = 0.29, 95% CI: 0.21–0.37, *P* < 0.00001, Figure 2A).

##### Performance status (KPS scores)

Nine trials (6, 10–16, 20) compared patients' performance status by KPS scores before and after the treatments. Meta-analysis showed that the KPS scores were significantly increased in CHM-WM group when compared with WM group (MD = 3.75, 95% CI: 0.85–6.65, *P* = 0.01, Figure 2B).

##### Tumor evaluation

Five trials (6, 8, 9, 15, 16) evaluated the tumor by Response Evaluation Criteria in Solid Tumors (RECIST) between two groups. Meta-analysis showed that the pathological change of tumor was significantly more stable in the CHM-WM group when compared with WM group (RR = 1.30, 95% CI: 1.01–1.67, *P* = 0.04, Figure 2C).

##### Immunologic function

Four trials (13, 15–17) evaluated the immunologic function by CD3, CD4, and CD8 counts before and after the treatments. Meta-analysis showed that CD4 counts level were significantly higher in CHM-WM group when compared with WM group (MD = 4.16, 95% CI: 1.25–7.06, *P* = 0.005, Figure 2D). CD3 and CD8 counts were not significantly different between CHM-WM group and WM group (WM) (MD = 3.74 CI: −0.43–7.91, *P* = 0.08, Figure 2E, MD = −076 CI: −4.07–2.54, *P* = 0.65, Figure 2F).

##### CA125

Four trials (10, 12, 13, 16) evaluated CA125 before and after the treatments. Meta-analysis showed that CA125 was significantly lower in CHM-WM group when compared with WM group (MD = −7.76, 95% CI: −12.57 to −2.95, *P* = 0.002, Figure 2G).

##### Quality of life

Quality of life was reported in 3 trials (6, 10, 16). Meta-analysis showed that there was no significant difference in quality of life between CHM-WM group and WM group (MD = 2.55, 95% CI: 0.01–5.10, *P* = 0.05, Figure 2H).

##### Three-year survival rate

Three-year survival rate was reported in 2 trials (19, 22). Meta-analysis indicated that the 3-year survival rate in CHM-WM group was significantly higher than in WM group (RR = 1.29, 95% CI: 1.06–1.57, *P* = 0.01, Figure 2I).

#### Safety

##### Gastrointestinal reactions

**Figure 3:**
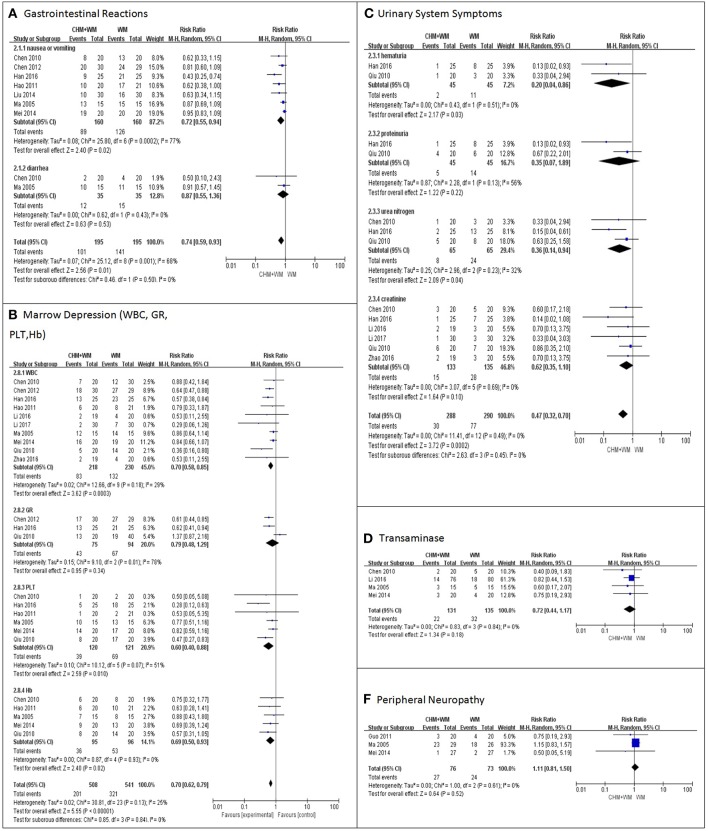
Meta-analysis on safety. **(A–F)** shown the comparisons and meta-analysis on safety between CHM-WM group and WM group. The I2 statistic described the percentage of total variation across studies that was due to heterogeneity rather than chance. CI indicated the confidence interval. Dichotomous data were presented as pooled Risk Ratio (RR) with 95% confidence intervals (95% CIs).

Gastrointestinal reactions (including nausea or vomiting, diarrhea) were recorded in 7 trials (6, 7, 10, 11, 14–16). Meta-analysis showed that the incidence of gastrointestinal reactions was significantly lower in CHM-WM group when compared with WM group (RR = 0.74, 95% CI: 0.56–0.93, *P* = 0.01, Figure 3A).

##### Bone marrow depression

Bone marrow depressions (parameters such as nausea or vomiting, diarrhea) were recorded in 10 trials (6, 7, 10–13, 15–18). Meta-analysis showed that the bone marrow depression in CHM-WM group was significantly lower than that in WM group (RR = 0.70, 95% CI: 0.62–0.79, *P* < 0.00001, Figure 3B).

##### Urinary system symptoms

Symptoms and markers in the urinary system including hematuria, proteinuria, urea nitrogen and creatinine, were recorded in 6 trials (6, 10, 12, 13, 18, 19). Meta-analysis results showed that incidence of urinary system symptoms in CHM-WM group was significantly lower than that in WM group (RR = 0.47, 95% CI: 0.32–0.70, *P* = 0.0002, Figure 3C).

##### Liver function

Liver function (parameters such as ALT) was recorded in 4 trials (6, 12, 15, 16). Meta-analysis indicated that there was no significant difference in liver function between two groups (RR = 0.72, 95% CI: 0.44–1.17, *P* = 0.18, Figure 3D).

##### Peripheral neuropathy

Peripheral neuropathy (such as loss of sensation, muscle weakness and atrophy, loss of tendon reflexes, and vasomotor symptoms) was recorded in 3 trials (9, 15, 16). Meta-analysis showed that there was no significant difference in peripheral neuropathy between two groups (RR = 1.11, 95% CI: 0.81–1.50, *P* = 0.52, Figure 3E).

##### Others

**Figure 4:**
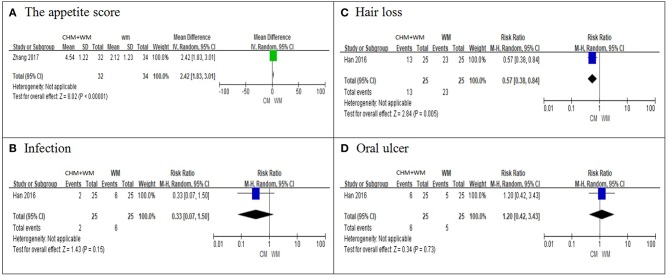
Other results. **(A–D)** shown the comparisons and meta-analysis on the appetite score, hair loss, infection and oral ulcer between CHM-WM group and WM group. The I2 statistic described the percentage of total variation across studies that was due to heterogeneity rather than chance. CI indicated the confidence interval. Dichotomous data were presented as pooled Risk Ratio (RR) with 95% confidence intervals (95% CIs), while continuous data were presented as Mean Difference (MD) with 95% CIs.

Additionally, one study (23) compared appetite score by daily intake between the two groups, and reported that the appetite score of the CHM-WM group (4.54 ± 1.22) was significantly higher than the score of the WM group (2.12 ± 1.23), (*P* < 0.05, Figure 4A). Another study (10) recorded the incidence rate of hair loss, infection and oral ulcer. It showed that the incidence rate of hair loss in CHM-WM group was significantly lower than that in WM group (*P* = 0.005, Figure 4B), but there was no significant difference in incidence rate of infection and oral ulcer between two groups (*P* > 0.05, Figures 4C,D).

## Discussion

In this study, we reviewed the efficacy and safety of CHM in the treatment of OvC after reduction surgery and adjuvant chemotherapy. A total number of 18 trials involved 975 patients were included, 488 patients in CHM-WM group, and 487 patients in WM group. Meta-analysis indicated that using CHM combined with WM improves the efficacy and safety of treatment on OvC patients.

The meta-analysis showed that the improvements of TCM syndromes and symptoms, KPS Scores, CD4, CA125, and 3-years survival rate in CHM-WM group were significantly better than WM group. These results implied that, compared with WM alone treatment, CHM combined with WM treatment can improve the symptoms and quality of life, consolidating the curative effects and alleviating the pain of OvC patients. There are no special symptoms in the early stage of OvC, and the methods for screening and early detection of OvC are still lacking. Therefore, most of the OvC patients were diagnosed at late stage, resulting in a poor prognosis (44). Although the adjuvant platinum-based chemotherapy is a therapeutically effective treatment after tumor debulking reduction surgery, adverse effects of chemotherapy, and high tumor recurrence are still a major problems for OcV patients (45). The application of CHM is extensive and profound, and there are a solid theoretical foundation and rich clinical experience in the treatment of cancer with CHM. Although CHM cannot inhibit the growth of tumors, CHM plays an important role in reinforcing healthy Qi, regulating the disharmony of Yin-Yang, Qi-Blood, and Zang-Fu, enhancing the patients' resistance, etc. These could be reflected by the improvement of performance status, TCM syndromes, and symptoms in the clinical trials.

Compared with WM group, the adverse effects including gastrointestinal reaction, marrow suppression, liver and kidney dysfunction and infection were significantly reduced in CHM-WM group. It implied that CHM combined with WM treatment could reduce the side effects caused either by the cancer itself or by the chemotherapy used in treating OvC. The toxic effects, medical complications and the poor quality of life are common, though surgery, radiotherapy, and chemotherapy have very good anti-cancer outcomes. Clinically, CHM combined with WM treatment not only provides higher clinical efficacy and longer survival time for patients, but also have therapeutically effects on alleviating and preventing the side effects of surgery, radiotherapy and chemotherapy. The mechanisms could be the formula was aimed at reducing the chemotherapy-induced side-effects, and some of the individual herbs included were also shown to have anti-oxidant and cytotoxic activities and they might also enhance cellular immunity

There are limitations in the study. Firstly, the methodology quality of the included RCTs was generally not high. Although all the studies claimed that randomization has been applied, only one study list the details of the randomized schemes and instructions. Six studies mentioned the application of “random number table” but without further details. The rest of studies only mentioned “randomization,” and we failed to get confirmation by contacting the original authors. Secondly, only one study reported blinding. We considered blinding was not carried out in most of the clinical studies due to clinical trial ethics on the treatment for cancer patients. Additionally, the included studies were mostly small sample sized. Thirdly, the CHM formula included studies which were different or not exactly the same. Based on TCM theory, personalized individual treatment plan should be applied according to the patient' s condition individually. So, our conclusion of this review is referring to the general concept of CHM, but not to individual CHM formula or individual herb.

## Conclusion

In conclusion, the results showed that CHM significantly improved symptoms and enhanced curative effects. CHM also showed the unique superior chemotherapy tolerance in quality of patient's life and minimal toxic and adverse effects due to chemotherapy. So, our review and meta-analysis have provided evidence on the efficacy and safety of CHM for ovarian cancer after reduction surgery and adjuvant chemotherapy, but rigorously designed and large-scale RCTs are still needed in the future.

## Data Availability

All datasets generated for this study are included in the manuscript and/or the Supplementary Files.

## Author Contributions

LL contributed conception and design of the study. LL, YL, RW, and QS organized the databases. RW, QS, FW, YL, HT, and WH performed the statistical analysis and prepared the figures and tables. LL, RW, QS, and FW wrote the first draft of the manuscript. YL, XL, TC, XW, HT, MZ, SZ, and YX wrote the sections of the manuscript. CW modified the English of the manuscript. All authors contributed to manuscript revision, read, and approved the submitted version.

### Conflict of Interest Statement

The authors declare that the research was conducted in the absence of any commercial or financial relationships that could be construed as a potential conflict of interest. The reviewer WL declared a shared affiliation, with no collaboration, with one of the authors, LL, to the handling editor at time of review.

## References

[1] Yang YBJ Li S Chen R Research and analysis on TCM therapy for ovarian cancer. J Liaoning Univ TCM. (2014) 5:121–4. 10.13194/j.issn.1673-842x.2014.05.043

[2] Song ZY Zhang G Zhang HL. Ovarian cancer. Xinjiang J Trad Chin Med. (2012) 1:90–2. Available online at: http://www.cnki.com.cn/Article/CJFDTotal-XJZY201201049.htm21532840

[3] Torre LA Trabert B DeSantis CE Miller KD Samimi G Runowicz CD. Ovarian cancer statistics, 2018. CA Cancer J Clin. (2018) 68:284–96. 10.3322/caac.21456 29809280 PMC6621554

[4] Lu HW Xie LL Lin ZQ Interpretation of “Ovarian cancer, version 1. 2016, NCCN clinical practice guidelines in oncology.” Chin J Pract Gynecol Obst. (2016) 8:761–8.

[5] Hao Y Zhang X TCM therapy for ovarian cancer research and analysis. J Pract Trad Chin Inter Med. (2011) 7:35–6. 10.13729/j.issn.1671-7813.2011.07.014

[6] Chen M Clinical research of warming the kidney and activating the blood methods combined with TP chemotherapy in the treatment of advanced ovarian cancer. Nanjing Univer Trad Chin Med. (2010). Available online at: http://cdmd.cnki.com.cn/article/cdmd-10315-2010245008.htm

[7] Chen LL Clinical research of warming the kidney and activating the blood methods combined with TP chemotherapy in the treatment of advanced ovarian cancer. Nanjing Univer Trad Chin Med. (2012). Available online at: http://cdmd.cnki.com.cn/Article/CDMD-10393-1012510164.htm

[8] Cheng QA Zhang QF Effect of Xiaozheng decoction combined with chemotherapy for treating patients with middle and terminal stage ovarian cancer who resistant to taxanes. J N Chin Med. (2015) 2:181–2.

[9] Guo W Clinical observation and nursing of endometrial and ovarian cancer treated by Chinese and Western Medicine. Henan Trad Chin Med. (2011) 06:689–91. 10.1016/j.jtcme.2019.07.001

[10] Han FJ Guo Y Tian M Sun R Clinical observation of Bushenxiaozheng method on the quality of life of postoperative ovarian cancer patients. Acta Chin Med Pharmacol. (2016) 2:67–9. 10.19664/j.cnki.1002-2392.2016.02.023

[11] Hao Y The clinical research on the soothing effects of TCM flavored shenlingbaizhu prescription towards the chemotherapy of postoperative ovarian cancer. Liaoning Univer Trad Chin Med. (2011).

[12] Li JR Effect of guizhi fuling capsule and earthworm combined with TC regimen on CA125 and quality of life of blood stasis type of ovarian cancer. Heilongjiang Univer Trad Chin Med. (2016). Available online at: http://cdmd.cnki.com.cn/Article/CDMD-10228-1016067436.htm

[13] Li Y Effect of self-made yangzheng guiling decoction combined with TP on ovarian cancerand T cell immune function. Heilongjiang Univer Trad Chin Med. (2017). Available online at: http://cdmd.cnki.com.cn/Article/CDMD-10228-1017164775.htm

[14] Liu HR Zhang SC Wang RC Feng WJ Clinical observation on effect of Shaofu Zhuyu decoction on reducing toxicity and synergism of postoperative chemotherapy for advanced ovarian cancer. In: The Co-operation of Chinese and Western Medicine Tumor Academic Conference. Guangzhou. (2014).

[15] Ma L Clinical study on YiQiJianPi and HuaYuJieDu method combined with TP regimen in the treatment of late-stage ovarian cance. Nanjing Univ Trad Chin Med. (2005). Available online at: http://cdmd.cnki.com.cn/Article/CDMD-10315-2006184268.htm

[16] Mei DY Clinical study on YiQiJianPi and JieDuSanJie method combined with TP Regimen in the treatment of late-stage ovarian cancer. Nanjing Univ Trad Chin Med. (2014). Available online at: http://cdmd.cnki.com.cn/Article/CDMD-10315-1015628716.htm

[17] Qiu LN Clinical and empirical study of Li Chong Sheng Sui Decoction interfere in ovarian cancer. Heilongjiang Univ Trad Chin Med. (2010). Available online at: http://cdmd.cnki.com.cn/article/cdmd-10228-1011037487.htm

[18] Zhao L Observe the clinical effects: changes of hemodynamic indexes by the treatment of a combination of “Guizhi Fuling capsules” with lumbricus and TC chemotherapy to the ovarian cancer patients who belong to blood stasis type and have accepted surgery. Heilongjiang Univ Trad Chin Med. (2016). Available online at: http://cdmd.cnki.com.cn/Article/CDMD-10228-1016055411.htm

[19] Jia F Analysis of effect of Traditional Chinese Medicine prescription combined with conventional Western Medicine treatment on advanced ovarian cancer. Strait Pharm J. (2017) 29:169–71. 10.3969/j.issn.1006-3765.2017.02.093

[20] Yi LJ Li SW He DM Clinical observation on ovarian cancer associated ascites treated by intraperitoneal infusion of modified Shenqi Baizhu powder combined with Interleukin-2. J Guangzhou Univ Trad Chin Med. (2017) 34:31–4. 10.13359/j.cnki.gzxbtcm.2017.01.008

[21] Mao ZJ Shen KP Zhu LM Zhu LM Yao Q Zheng JL Effect of modified CHM formulae on the quality of life of ovarian cancer patients. Chin J Woman Child Health Res. (2017) S1:656–7.

[22] Xu Y Effect of Traditional Chinese Medicine combined with TP chemotherapy on quality of life of patients with advanced ovarian cancer. Chin J Med Guide. (2016) 18:1144–5. Available online at: http://www.cnki.com.cn/Article/CJFDTotal-DKYY201611034.htm

[23] Zhang LY The Effect of Chinese Herbal application and acupoint massage on the loss of appetite in patients with ovarian cancer during chemotherapy. J Qilu Nurs. (2017) 23:77–8. 10.3969/j.issn.1006-7256.2017.10.035

[24] Han SY Zhu H Jia CR Wang XX Clinical study on fuzhengyiliu decoction for reducing toxicity and enhancing efficacy and anti-tumor. J Emer Trad Chin Med. (2007) 16:285–7. 10.3969/j.issn.1004-745X.2007.03.018

[25] Wang XP Clinical experience of TCM combined with chemotherapy on advanced ovarian cancer. Asia-Pacific Trand Med. (2012) 8:84–5. 10.3969/j.issn.1673-2197.2012.12.047

[26] Pan L Gao H Xin XR Yin DF Clinical observation on the prevention of peripheral neuropathy induced by paclitaxel chemotherapy by internal and external application of traditional Chinese medicine. Inner Mongolia J Trad Chin Med. (2012) 3:28 10.16040/j.cnki.cn15-1101.2012.03.204

[27] Zhao D Deng J Zhu YT Shi DH Systematic review of TCM combined with TP on advanced ovarian cancer. Strait Pharm J. (2015) 27:71–3. Available online at: http://www.cnki.com.cn/article/cjfdtotal-haix201501032.htm

[28] Chen JJ The study of clinical efficacy evaluation and survival analysis of combined traditional Chinese and western medicine on advanced ovarian cancer. Zhejiang J Trad Chin Med. (2012) 47:751–2. 10.3969/j.issn.0411-8421.2012.10.040

[29] Chen J Zhang ZH Zhou J Cao SL Chen GY The study of Peripheral blood T lymphocyte subsets oh ovarian cancer. Chin J Pract Gynecol Obstet. (1993) 9:353–5. Available online at: http://www.cnki.com.cn/Article/CJFDTotal-ZGSF199306022.htm

[30] Zhang J Cheng JX Shan BE Shi WC The study of compound Chinese medicine on proliferation of peripheral blood mononuclear cells and TNF-α level on ovarian cancer patients. Clin Focus. (2005) 10:580–1. 10.3969/j.issn.1004-583X.2005.10.029

[31] Pan TH Fan QY The study of surgery, chemotherapy and traditional Chinese medicine on primary ovarian cancer. J Anhui TCM College. (2004) 23:15–7. 10.3969/j.issn.1000-2219.2004.04.007

[32] Chen J Wang XH Chen LS Lin L Chen LL Clinical observation on 27 cases of ovarian cancer treated by nourishing and expelling pathogenic factors. Fujian J TCM. (2011) 42:14–6. 10.13260/j.cnki.jfjtcm.010108

[33] Wang J Sui LH Lou G Xu F The study of element on ascites of ovarian cancer. Acta Chin Med Pharmacol. (1991) 1:35–6. 10.19664/j.cnki.1002-2392.1999.01.031

[34] Wang BS Liu XF Wang LL Li CY Ding RL Wang T The study of TCM on advanced ovarian cancer with refractory ascites. Chin J Inform Trad Chin Med. (2011) 8:78–9. 10.3969/j.issn.1005-5304.2001.09.049

[35] Wang HC Zhao YL Clinical analysis of tuyuan decoction on ovarian cancer. J Liaoning Univ TCM. (2008) 8:110–1. 10.13194/j.jlunivtcm.2008.08.112.wangkc.062

[36] Zhang W Wang MX Guo YF Tan XY The study of matrine on perioperative period of ovarian cancer patients. Chin J Lab Diag. (2014) 18:1290–1. Available online at: http://www.cnki.com.cn/article/cjfdtotal-zszd201408030.htm

[37] Yu JF Clinical study on ovarian tumour treated with fuzhengxiaoliu decoction combined with chemotherapy. Heilongjiang Univ Trad Chin Med. (2005). Available online at: http://cdmd.cnki.com.cn/article/cdmd-10228-2005120574.htm

[38] Cheng K The clinical research on the treatment of ovarian tumor by Lichongshengsuiyin combined with chemotherapy. Heilongjiang Univ Trad Chin Med. (2010). Available online at: http://cdmd.cnki.com.cn/article/cdmd-10228-1011037600.htm

[39] Liu Y Clinical study on oophoroma after treated with Fuzhengquyu decoction combined with chemotherapy. Heilongjiang Univ Trad Chin Med. (2008). Available online at: http://cdmd.cnki.com.cn/article/cdmd-10228-2008179650.htm

[40] Xia J Efficacy analysis of LCSSY on attenuation of postoperative chemotherapy in patients with ovarian cancer. Heilongjiang Univ Trad Chin Med. (2017). Available online at: http://cdmd.cnki.com.cn/Article/CDMD-10228-1017145170.htm

[41] Chan KKL Yao TJ Jones B Ma FK Leung CY Lau SK. The use of Chinese herbal medicine to improve quality of life in women undergoing chemotherapy for ovarian cancer: a double-blind placebo-controlled randomized trial with immunological monitoring. Ann Oncol. (2011) 22:2241–9. 10.1093/annonc/mdq749 21355071

[42] Li Y Zhang T Guo YP Liu L Zhu JH Zhao Y Effect of cantharidin sodium vitamin B6 combing with PT therapy on the lymphatic growth factor of ovarian cancer tissues. J Hainan Med Univ. (2015) 21:1701–4. 10.13210/j.cnki.jhmu.20151021.003

[43] Piao BK Wang YX Xie GR Mansmann U Mathess H Beuth J. Impact of complementary mistletoe extract treatment on quality of life in breast, ovarian and non-small cell lung cancer patients. a prospective randomized controlled clinical trial. Anticancer Res. (2004) 24:303–10. 10.1007/BF00725830 15015612

[44] Shen WJ Dai DQ Advances of epigenetics in ovarian cancer. Chin J Cancer Prev Treat. (2010) 14:790–4. 10.16073/j.cnki.cjcpt.2007.10.021

[45] Yang Y Clinical study of docetaxel combined with platinum for the treatment of 58 cases recurrent ovarian carcinoma. Chin J Cancer Prev Treat. (2008) 15:1176–7. 10.16073/j.cnki.cjcpt.2008.15.016

