# Supplementary material for: Efficacy and Safety of Chinese Herbal Medicine on Ovarian Cancer After Reduction Surgery and Adjuvant Chemotherapy: A Systematic Review and Meta-Analysis
Source: Front Oncol. 2019 Aug 16;9:730. doi: 10.3389/fonc.2019.00730 (PMC6706872; doi:10.3389/fonc.2019.00730)
Supplement: Supplementary file 1 [file Table_1.docx]

| Supplementary Table: Summary of all meta-analysis | | | | | |
| --- | --- | --- | --- | --- | --- |
| Outcomes | No.of Studies | No.of Participants(T/C) | Analysis Results (95%CI) | P value/I^2^(%)for Heterogeneity | Analytical model |
| Syndromes and Symptoms | 10 | 235/235 | RR=0.29 (0.21~0.37), P＜0.00001 | P=0.82/I^2^=0 | fix-effect |
| Karnofsky Performance Status Scale | 9 | 418/422 | MD=3.90 (1.22~6.58), P=0.004 | P＜0.00001/I^2^=91 | random-effects |
| Response Evaluation of Solid Tumors | 5 | 113/113 | RR=1.30 (1.01~1.67), P=0.04 | P=0.31/I^2^=16 | fix-effect |
| CD4 | 4 | 170/170 | MD=4.16 (1.25~7.06), P=0.005 | P＜0.00001/I^2^=91 | random-effects |
| CD3 | 4 | 170/170 | MD=3.74 (-0.43~7.91), P=0.08 | P＜0.00001/I^2^=95 | random-effects |
| CD8 | 4 | 170/170 | MD=-0.76 (-4.07~2.54), P=0.65 | P＜0.00001/I^2^=90 | random-effects |
| CA125 | 4 | 188/190 | MD=﹣7.76 (-12.57~-2.95), P=0.002 | P=0.0001/I^2^=76 | random-effects |
| Quality of Life | 3 | 180/176 | MD=2.55 (0.01~5.10), P=0.05 | P=0.0003/I^2^=79 | random-effects |
| Three-year Survival Rate | 2 | 82/82 | RR=1.29 (1.06~1.57), P=0.01 | P=0.68/I^2^=0 | fix-effect |
| Gastrointestinal Reactions | 7 | 195/195 | RR=0.74(0.59~0.93), P=0.01 | P=0.001/I^2^=68 | random-effects |
| Marrow Depression | 10 | 508/541 | RR=0.70(0.62~0.79), P＜0.00001 | P=0.13/I^2^=25 | fix-effects |
| Urinary System Symptoms | 6 | 288/290 | RR=0.47(0.32~0.70), P=0.0002 | P=0.49/I^2^=0 | fix-effect |
| Liver function | 4 | 131/135 | RR=0.72 (0.44~1.17), P=0.18 | P=0.84/I^2^=0 | fix-effect |
| Peripheral Neuropathy | 3 | 76/73 | RR=1.11 (0.81~1.50), P=0.52 | P=0.61/I^2^=0 | fix-effect |
